# The Hospital Nursing Supplement

**Published:** 1893-07-22

**Authors:** 


"The Hospital" Nursing Mirror.

Being the Extra Nursing Supplement of "The Hospital" Newspaper.

[Contributions for this Supplement should be addressed to the Editor, The Hospital, 428, Strand, London, W.C., and should have the word "Nursing" plainly written in left-hand top corner of the envelope.]

### News from the Nursing World.

### A ROYAL VISIT.

The Queen of Denmark visited the Hospital for Sick and Incurable Children in Cheyne Walk last week, and went through the wards, accompanied by H.R.H. the Princess of Wales and her daughters, the Princesses Victoria and Maud.

### A PLEA FOR SICK PAUPERS.

Amusing books and illustrated magazines are as much part of our annual holidays as the railway rug and the fishing-rod of the adult, or the sand shoes, buckets, and spades of the youngsters. The ultimate destination of the novels and story books is generally a matter of indifference to the purchasers when they have served their immediate purpose. We beg to suggest to such of our readers as indulge themselves in books or magazines on long journeys that they may confer great pleasure on the inmates of workhouses, especially those in the sick wards, by forwarding the discarded literature to the nearest institution, addressed, "For the Use of the Patients."

### ARE YOU REGISTERED, NURSE?

Certain members of the public are apt to use a word that is new to them without appreciating its meaning. Nurses are sometimes asked by their patients whether they are registered, under the impression that there must be a chartered register for nurses, and that every registered nurse is equal to, if not better than, a registered doctor. There is, of course, no chartered register for nurses, and if there were, registered nurses would not be doctors, nor would any sensible woman permit this idea to prevail for one moment, so far as she was concerned. Such an idea must be fatal to her progress, as it brings her into direct conflict with the members of the medical profession, and must materially interfere with her earnings. Any nurse who is asked this question would be well advised to produce her certificate of training, and to reply: "I am a trained nurse, and here is my certificate." It is imperative that medical practitioners and members of the public, as well as nurses, should understand this question in all its bearings.

### GRIEVANCES.

"How is your sister?" asked an old friend. "Quite well, thanks, and quite happy, for she's got a grievance," replied one who had studied human nature successfully. But so-called "grievances" do not make everybody happy, although there is a universal tendency to embrace, to encourage, and to enlarge upon real and imaginary ones. Somebody or other is constantly complaining of something, and a correspondent last week boldly headed her communication "A Grievance," but as she fails to comply with our invariable rule that all communications must be accompanied by the writer's full name and address, we cannot help her to clear away, or even to foster her own pet variety.

### WHAT BECOMES OF THE FLOWERS?

West-end balconies are still bright and attractive with flowers and trailing plants, pleasant alike to owners and to onlookers. In a short time all will be changed, the fine houses will be shut up, because "everybody is out of town," and then the flowers will die, and the ornamental shrubs will follow, and dried stalks and withered leaves will sadden the passers-by till "the family" is again in residence. It seems a pity that it does not occur to anybody to give away all the pots before the fading begins. It would be easy enough to transfer them to institutions or to poor districts. There are many so-called "charities" which are never visited, and seldom thought of, even by people who are quite familiar with hospital wards, and by them these discarded plants would be tenderly cared for and valued.

### WHERE AND WHEN?

One of the first questions asked just now by our friends is "When are you going away?" or else "Where are you going for your holiday?" We hope that all our readers will enjoy a pleasant change of scene, and that they will return rested and refreshed to their daily duties. Where they go and when they start is a matter of purely personal interest, but in one phase of holidays The Hospital ventures to claim a hearing. "Doing nothing" proving absolutely impossible to any intelligent woman, and change, not cessation of occupation, being the truest recreation, we again advance the claims of the adult sick poor in hospital wards. Last Christmas we had some beautiful clothes sent to us for distribution, and they were received with exceeding joy by those to whom gifts are unfortunately too rare to lose the charm of novelty. This year we will gladly receive as many more as our readers will make for us, and we think it is probable that our friends will enjoy their holidays none the less if they return home with some completed and serviceable garments for the sick poor packed away in their trunks. A list of the prizes offered and garments chiefly required will shortly appear in our columns.

### "SOMEONE HAD BLUNDER'D."

If the blunder of a moment may sometimes have no bad results, at others it becomes little short of a crime. In history the latter is naturally the only class of error which comes before us, and perhaps few lines of Tennyson's are more pathetically significant than those which occurred in the "Charge of the Light Brigade," "tho' the soldier knew someone had blunder'd." It is not only in the army and the navy that mistakes occur, they are pitifully frequent elsewhere, although the disastrous results are less widely known. We apprehend unhappy events must surely ensue when an untrained ward maid is suddenly made a nurse in a cottage hospital, and at twenty-three years of age is already in charge of twenty patients. Surely someone has blundered in making such an appointment as this? Twenty-three is a very suitable age for a wardmaid, but it is far too young for anyone to be placed in a position of such serious responsibility as attaches to a nurse. When the inevitable disaster arrives, public opinion will fail to discover that ignorance and youth are "extenuating circumstances."

### MALE NURSES.

Where can a man be thoroughly trained in sick nursing? The question is repeatedly asked, and always receives the same answer, for none of the general hospitals or infirmaries appear able to grant regular instruction to male probationers. In institutions for mental diseases and chronic or nervous ones men are employed, but this gives them no training in general nursing or surgical work. Knowing that skilled men-nurses are the only ones suited to certain male patients and to various diseases, we shall be glad to hear of English institutions taking up the subject of their adequate training.

### MUST THE WARD BE CLOSED?

It would be difficult to say exactly how much good work has been done in the Pendlebury Children's Hospital. Thousands of little patients have been cared for within its walls during the sixty-five years which have elapsed since its foundation. In the course of 1892 nearly 1,300 cases were treated inside, and over 1,000 outside the hospital. At present this deserving and greatly valued institution is suffering from the universal complaint of impecuniosity, and it is possible that one of the wards will have to be closed unless funds are speedily forthcoming. This would be a very serious evil, for even now patients from all the surrounding districts are awaiting admission. Surely Manchester will not suffer the carrying out of this threat to close one of the five wards now open? It does not take long to shut up a ward, but a row of empty hospital cots is one of the saddest sights in the world, for their occupation would mean relief and often recovery to so many ailing children. It is bad enough when "no room" has to be the answer to the anxious mother whose little one is perishing for lack of skilled attention and appliances; but "wards closed for want of funds" is a far worse announcement, and one which ought to be impossible. If every parent of a healthy child in Manchester and the neighbourhood would let his thankoffering for this blessing take the form of a donation to the Children's Hospital, there would be rejoicing in many a poor man's dwelling.

### DISTRICT NURSES IN AMERICA.

An association was organised at Chicago in 1889 to provide trained nurses for the sick poor in their own homes. Under the title of Visiting Nurses nine skilled workers have not only attended personally to the sick, but they have given practical lessons to the friends of the patients in every branch of home nursing. Three emergency nurses are also employed, and these give attention to special cases, and are allowed to remain for a day, a night, or longer if required. They are also sent to contagious diseases, which they manage so intelligently that one case of infectious illness is not followed by others, however crowded the tenement.

### MISS NIGHTINGALE'S PAPER.

The announcement that Miss Nightingale would contribute a paper to the International Congress of Nursing raised great expectations in the minds of our American sisters. Knowing her keen and progressive interest in all that concerns them and their work, they thoroughly understood the weight which would attach to her opinions. No one can fail to see that Miss Nightingale, although debarred by delicate health from taking a personal part in public functions, has still a wise judgment, and large-minded grasp of the numerous subjects submitted to her. Rather, we might say, her retired life enhances the value of her words; she can decide dispassionately on the masses of facts laid before her, without the interruptions of any embarrassing personal influences. With expectations highly raised to hear what Miss Nightingale's paper might contain, great dismay ensued on its non-arrival by the first day of the Congress at Chicago; however, it eventually came safely to hand, and was read by Miss Hampton to a most attentive audience on Friday, June 16th. It excited a great deal of attention and interest, and we propose to publish it in full later on, that English as well as American nurses may benefit by it.

### ON LAND AND SEA.

The new steam launch which has been purchased by some friends and presented to the Mission to Deep Sea Fishermen, has been named the "Princess May." The vessel has gone to Labrador, and is specially suited for the purpose of conveying sick or wounded seamen, a little adaptation having rendered it possible to place an injured man direct on to a full length settle, wide enough for his back, making it an easy matter to convey him to the hospitals or to the "Albert." There are now eleven ships engaged in the work of the Mission. Our readers will understand how urgently this hospital accommodation is needed for the men engaged in this dangerous calling. In the North Sea fishing fleets alone 20,000 men and boys are employed, and as the cruises last eight weeks it is essential that adequate medical attendance should be supplied to them during these periods. Two fully trained and experienced hospital nurses have accompanied the medical mission to Labrador this year, Miss Cecilia Williams and Miss Carwardine, both of the London Hospital. Visitors to the East Coast would do well to remember that their personal services, as well as gifts, will help to make a success of the floating bazaars, announced in another column.

### A NOBLE EXAMPLE.

For the Women's Hospital, Melbourne, we are told that the handsome sum of £6,000 has been raised by "a week of self-denial." Each person who voluntarily abstained from some usual indulgence gave the amount saved. Leaving off butter was a favourite plan, especially with the young, and one gentleman by using post-cards instead of stamps saved five shillings. If such a sum could be raised in Melbourne, what might not be done in some of our large English towns?

### SHORT ITEMS.

There are over two hundred women doctors practising within the boundaries of Chicago.—An excellent Amateur Photographic Exhibition was recently held at Melbourne in aid of the funds of the Children's and the Austin Hospitals.—A third district nurse has been engaged by the Gateshead Nursing Association, and a house has been taken for a nurses' home.—They are about to establish a Nurses' Home in Ceylon to provide the European residents with trained attendants in sickness.

### THE DISABLED NURSE.

We have pleasure in acknowledging 5s. received from F. S. this week for the disabled nurse, and 2s. from Policy 1,281, making a total of £14 12s. 6d., of which £14 7s. 6d. has been already handed to her. Our subscription list is now closed, and we cordially thank all those who have helped in this matter.

### Sanitation for Nurses.

By a Sanitary Inspector.

### XIV.—HEAT AS A DISINFECTANT.

One of the most certain disinfectants is heat, and especially moist heat or steam. For the following details we are indebted to Dr. Parson's report to the Local Government Board, as condensed by Dr. Wynter Blyth. By means of most careful experiments with dry heat, it was found "that spores of anthrax lost their vitality after exposure for four hours to a temperature a little over that of boiling water, viz., 212—216° F., or for one hour at a temperature of 245° F., non-spore-bearing anthrax and the bacilli of swine-fever were rendered inert by exposure for an hour to a temperature of 212—218° F., and even five minutes' exposure to this temperature sufficed to destroy the vitality of the former and impair that of the latter." Dr. Klein made some experiments with boiling water, and proved that anthrax spores were destroyed by exposure to boiling water for one minute only, whilst his experiments with steam convinced him that all form of contagion were effectually des troyed by exposure to steam at 212°F. for only five minutes. As a result of a large number of experiments with both dry and moist heat, it has been proved that the former is a far less effectual disinfectant than the latter, because it has not the same power as steam possesses of penetrating completely. Moreover, articles can be exposed to a higher temperature with steam than they can with dry heat, since the risk of the latter is that the articles will be scorched, and most articles are injured, if not destroyed, if exposed for long to a dry temperature of 255° F. whereas comparatively few articles are seriously injured by steam. The value of steam as a disinfectant for such articles as bedding is very marked—these articles are readily penetrated by steam, whereas it is most difficult, if not impossible, to secure that they shall be thoroughly penetrated by dry heat, because such things as pillows, bedding, and blankets are very bad conductors of heat, and as the infection is not confined to the exterior, it is very important that the interior of all such articles should be exposed to the proper degree of heat. We will give two tables drawn up by Dr. Wynter Blyth to show the results of dry heat and steam respectively on pillows of various sizes and materials.

Dry Air.

| Kind of Pillow. | Weight in lbs. | Temperature to which exposed. | Time in hours. | Temperature in centre. | Result. |
|---|---|---|---|---|---|
| | | Deg. F. | | Deg. F. | |
| Feather ......... | 5 | 312 | ½ | 140 | Scorched |
| Flock ............ | 4 | 280 | 2 | 147 | ,, |
| Feather (tight) | 3 | 344 | 4 | 138 | ,, |
| Feather (loose) | 3 } | above | 1 | 275 | ,, |
| Flock ............ | 6 } | 275 | 2 | 138 | ,, |
| Flock ............ | 4¼ | 318 | 1 | 136 | ,, |
| Flock ............ | 3 | 250–260 | 5½ | 227 | Not scorched |
| Horsehair ...... | 2 | 276 | 1 | 149 | ,, |
| Feather ......... | 5 | 290 | 1 | 122 | ,, |

Steam.

| Kind of Pillow. | Weight in lbs. | Temperature to which exposed. | Temperature in centre. | Time. | Pressure of Steam. |
|---|---|---|---|---|---|
| | | Deg. F. | Deg. F. | | |
| Feather (tight) | 6½ | 251 | 185 | ½ hour | 15 lbs. |
| Feather ...... | 3¼ | 251 | 237 | ½ hour | — |
| Flock ......... | 3¼ | 240 | 171 | 2 min. | 10 lbs. |
| Flock ......... | 3½ | 240 | 234 | 10 min. | 10 lbs. |
| Feather ...... | 3½ | 240 | 234 | 10 min. | 10 lbs. |
| Feather ...... | 2¼ | 212 | 182 | 10 min. | — |
| Feather ...... | 2¾ | 252 | 251 | ½ hour | 22 lbs. |

The following table will show how very slowly dry heat will penetrate a woollen article which is folded in several layers; the experiment was tried on a blanket:—

| Duration of Exposure. | 2 Layers. | 4 Layers. | 6 Layers. | 12 Layers. | 18 Layers. |
|---|---|---|---|---|---|
| | Deg. F. | Deg. F. | Deg. F. | Deg. F. | Deg. F. |
| 4 hours......... | 220 | 206 | 190 | 162 | 139 |
| 6 hours......... | 226 | 214 | 208 | 174 | 153 |
| 8 hours......... | 230 | 221 | 215 | 196 | 182 |

An experiment made by Dr. Whitelegge on a blanket folded in sixteen layers exposed to steam proved that the temperature in the innermost folds reached 212° F. in 17 minutes. From these tables we see that steam most nearly fulfils the four conditions in a thorough disinfectant: "1, That it shall be capable of killing germs and their spores; 2, that it shall be applied to every part; 3, in sufficient strength; 4, for a sufficient time."

"When steam penetrates into the interstices of a cold body it undergoes condensation in imparting its latent heat to the body. When condensed into water it occupies only a very small fraction of its former volume. To fill the vacuum thus formed more steam presses forward, in its turn yielding up its heat and becoming condensed, and so on until the whole mass has been penetrated. On the other hand, hot air in yielding up its heat undergoes contraction in volume, it is true, but only to a very small extent as compared with that undergone by steam in condensing into water. Thus air at 250° F. in cooling to 50° F. would contract to $\frac{5}{7}$ of its previous volume. The heat which is evolved in the moistening of a dry porous substance is another source of advantage on the side of steam because (1) when a liquid wets a solid there is disengagement of heat; (2) when a solid absorbs a liquid there is disengagement of heat. The amount of heat liberated in the incorporation of a liquid into the substance of a solid is much greater than that liberated by mere superficial wetting; this is owing to the multiplication of points of contact. In proportion as a wetted substance is finely divided the number of particles which are moistened and take part in the evolution of heat is multiplied, while the number is diminished of those which not being wetted do not give out heat, but absorb it at the expense of others."

The penetrative power of steam is much increased by pressure, and it has further been proved that this penetrative power of steam is very greatly increased when the pressure is taken off and then reapplied from time to time. "A pressure of steam 15 lbs. to the square inch above that of the atmosphere compresses the air in the interstices of the material to half its original volume, and thus the space originally occupied with cold air becomes partly occupied with hot steam; steam partly mixed with air occupying the interstices near the surface, and air those in the centre. Equilibrium of pressure having been obtained, the interchange of air and steam particles and of temperature would take place comparatively slowly. On releasing the pressure, the air again expands to its original volume, and thus becomes mixed with the steam; and on the pressure being reapplied, the steam is forced further into the interstices."

Dr. Parsons classifies the various injuries to which things subjected to dry heat or steam are liable under seven heads, as follows: 1. Scorching or partial decomposition of organic substances by heat. At first showing itself by change of colour, texture, and weakening of strength. 2. Over drying, whereby things are rendered brittle. 3. Fixing stains so that they cannot be removed. 4. Melting such substances as wax, varnish, &c. 5. Altering colour, gloss, &c., of dyed and finished goods. 6. Shrinking and felting of woollen goods. 7. Wetting. Fortunately, however, disinfection by heat, though so excellent, is not the only method at our command, and by a judicious selection of a disinfectant, so long as it is really effectual, we can reduce to a minimum the injury to the things to be disinfected.

### The "Pall Mall Gazette" and the London Hospital.

The *Pall Mall Gazette* of the 18th inst. contains an article entitled "The Truth About the London Hospital," which is avowedly written in response to an invitation published in our columns. The article is stated to be written by a special commissioner who paid thirteen guineas to enter the London Hospital as a paying probationer for three months' training as a nurse. It is remarkable that the greater portion of the article contains practically the old charges against the Matron and her department which were dealt with by the Lords' Committee, and the animus of the writer is sufficiently indicated by the fact that she describes Miss Luckes as "that person." The only new point dealt with is the waste of good food, as to which we have to point out that during our recent inspection we were in the wards and kitchen at the time the dinners were being served, and, so far as our observation went, the food supplied was carefully distributed without waste. As to the surplus bread, we found that it was carefully collected and partly utilised for puddings, and that so much of it as was not wanted for this purpose was sold to a contractor, who removed it each day.

Can our contemporary the *Pall Mall Gazette* have been hoaxed, or, at least, seriously misled? It certainly looks like it, for the article states "that having read the appeal" which appeared in The Hospital of June 3rd last, the writer "resolved to enter the London Hospital as a nurse." That is, she resolved to enter as a nurse, did so enter, and has written her article as the result of the information so obtained. Now this article purports to be a description of many parts of the hospital and of its managament, and makes statements which could not possibly be learned by a paying probationer of a few days' residence. But, if it be true that our appeal induced the writer to go as a paying probationer to the London Hospital, she could not have entered it earlier than July 3rd, for it appears from inquiries we have made that no paying probationer has been admitted to the hospital between the date when our first article appeared (June 3rd), and the present date (19th July) with one exception. The paying probationer who constitutes the exception entered the London Hospital on July 3rd last. It is therefore important to ask the editor of the *Pall Mall Gazette* to promptly publish the date when his special commissioner became a paying probationer at the London Hospital, and the date when she left it? If she entered the hospital on July 3rd, where did she gain her information from, seeing that her first article appeared in the *Pall Mall Gazette* on the 18th July, and that it is humanly impossible that any paying probationer could have seen the things which she describes within the fortnight mentioned? In these circumstances, is not the reputation of the new management of the *Pall Mall Gazette* for the moment at stake, rather than that of the London Hospital?

The publication of this series of articles in the *Pall Mall Gazette* should in any case serve one useful purpose, as we presume the Editor will insist upon his commissioner supporting the statements contained in them at any public enquiry which the London Hospital Committee may be wise enough to institute without delay. If this step be taken, and the *Pall Mall Gazette*, with its usual fairness, publishes the evidence given, we feel certain that the Editor will in the end admit that the zeal of his commissioner has sadly outrun her discretion. Meanwhile, we must disclaim the statement that we hold a brief for the London or any other hospital, although it is our privilege to endeavour to secure fair play for the whole of the medical institutions without fear or favour. We would further point out that the articles which led the *Pall Mall Gazette* to send a commissioner into the London Hospital appeared in The Hospital so recently as June 3rd and 10th. Remembering this, and in face of the facts stated already, although we purposely defer dealing with the statements in detail until the series of articles is completed, another fact is noteworthy. Much of the "copy" in the *Pall Mall* article bears a close resemblance to that which appears in the Blue books, and the correspondent further reiterates a statement which the Chairman of the London Hospital at a recent meeting of the Governors declared to be false, viz., that probationers who are only half trained are sent out by the hospital authorities to care for private patients. We await the further articles with interest, but incline to the belief, after carefully perusing the one already published, that the Editor of the *Pall Mall Gazette* would have been well advised had he acted upon our suggestion that he should go himself to the London Hospital and there ask his correspondent to show him what she had discovered before publishing anything on the subject.

### Educational Standards for Nurses.

By Miss Isabel A. Hampton, Superintendent of the Johns Hopkins Hospital, Baltimore, Maryland.

### II.—PRESENT EDUCATIONAL ADVANTAGES AND DEFICIENCES.

Let us then consider (1) what is the present standard for the trained nurse; (2) what are her educational advantages? and (3) in what ways is she deficient? The object of schools for nurses is primarily to secure to the hospital a fairly reliable corps of nurses, and in order to insure a continuous source of supply, such schools are established and certain inducements are offered to women to become pupils in them. These inducements are set forth in the circulars of general information published by each school, and when one compares these circulars, the teaching methods of no two schools will be found to be alike, all varying according to the demands of the various institutions and their several authorities. Each school is a law unto itself. Nothing in the way of unity of ideas or of general principles to govern all exists, and no effort towards establishing and maintaining a general standard for all, has ever been attempted. Some institutions consider that a two years' course of instruction is essential; others place it at a year and a half, and others again at a year. In England a few schools insist upon three years. The hours of daily work also differ widely, some requiring from their pupils nine hours a day of active service; others as high as twelve and thirteen hours. The theoretical instruction is usually given outside of the nine hours' work, and it is difficult to speak definitely upon this subject, as the length of such a course, the subjects, and the extent to which they are taught, are again dependent upon the opinion upon this matter of the governing body of that particular school. We also find no general rule governing the special attainments or degree of education required from the women who present themselves as candidates. On the contrary, a woman who has been refused by or dismissed from one school for lack of education, dishonourable conduct, inefficiency, &c., frequently gains admittance into another, where the authorities have not so high, if any, standard required from those whom they accept.

#### The Value of the Diploma.

But notwithstanding all these differences, each woman who graduates from any of these schools, usually has the high-sounding name of diploma presented to her, and henceforth she is known as a trained nurse, with no other insignia to show what amount of knowledge or fitness she really does bring to her work. In fact, it is no unusual occurrence for schools to graduate nurses whom they at once, when relieved from their presence in the hospital, refuse to recommend or sustain in their work. A "trained nurse" may mean anything, everything, or next to nothing, and with this state of affairs the results are far from what they should be, and public criticism is frequently justly severe upon our shortcomings, or else is content with superficiality where like meets like. This criticism falls both upon the woman herself and upon the institutions which she represents. Sometimes the one only, in others both deserve censure. Can a woman be expected to give properly a hypodermic injection to a patient if her school has never taught her how this is to be done? The school and not the nurse is to be blamed for her ignorance in such a case. Or, again, abscesses may follow such injections unless the nurse has been taught the practical significance of antisepsis. Or, again, can she be expected to have at her fingers' ends the principles and practice of invalid dietary when she has never been practically taught such a thing. And when a really capable woman realises that she does not know enough to do her work sufficiently well to honourably receive the full compensation of a skilled nurse, and that she is not worthy of such responsibility, and if she is willing to give up more time and labour to go once more in a hospital where she may be really taught what she wants to know, where shall we find one capable school willing to take her? For we wish to mould our own fresh material, not being Michael Angelos to make Davids out of others' failures.

### A Great Want to be Supplied.

Sadly frequent, too, are these requests made to the authorities of our larger schools, and in most instances they are the fruits of the systems prevailing in our smaller hospitals and specialty hospitals. Such places are legitimate enough in their way, and indeed many are very necessary, still the mere fact that they are hospitals in no way justifies them in establishing training schools for the sake of economy, and accepting as their pupils women who, perfectly ignorant of what they need, go to them and give up a year, or two years, of precious time and then find that their education has been thoroughly inadequate to meet what is expected of them by the public. We cannot but feel that a real injustice is often done in such cases. If the nurse had gone into that hospital as a philanthropist it would be different, but she went there for the purpose of acquiring a certain kind of education. Again, these small hospitals not having the same number to select from as the larger schools, are apt, and in fact do, take women who, not being intellectually capable of comprehending the high calling into which they have been admitted, tend to lower the standard to which we are striving to attain. As an instance of such small hospitals which have come to my notice while writing on this subject, I have in my mind one, the superintendent of which informed me that their hospital contained thirty beds, and that they had a training school of eleven nurses. But the most pernicious of small hospitals is the specialty hospital or private sanatorium, which owes its existence solely to the desire of the owner to make money for himself, and in which a training school is organised for the sake of securing cheap nursing, with an utter disregard for the interests of the women employed. One doctor, who owns such a hospital with twenty-five beds, has sixteen nurses, who are given a two years' course in this particular specialty, but four of these nurses are actually sent to his private patients at twenty-five dollars per week (£5), this money going to the hospital, while the nurse receives sixteen dollars (£3 4s.) per month.

### Should Probationers be Sent to Private Houses?

This brings us to the question of the advisability of sending nurses out of the hospital into families during the second year of their training. It is true that such a procedure materially assists in the maintenance of the hospital, and to some hospitals it is very necessary, but is it exactly what should be done in the best interests of the education of the nurse? The majority of schools make the statement in their general circular that they reserve the right to send their pupils out to private duty during their second year in order to help to meet the expense of their maintenance and education. This may have been all well and good in the beginning of such schools, when hospitals were not so numerous in the land, and when the question of supporting them was a more serious difficulty than now. The additional expense of maintaining a school was not to be thought of, and it was thus necessary to appropriate some of the pupils' time towards providing an income. But now that wealthy philanthropists and societies are erecting hospitals of all kinds, they should see to it that the question of maintaining a nursing corps is provided for, instead of expecting the nurses to do philanthropic work by earning money to support the hospital at the sacrifice of their own education.

### The Value of Training Schools.

As a matter of fact, the services rendered by a good training school to a hospital, are sufficient to warrant the expenses incurred by the school, for, in any case, a certain amount of work has to be performed, and for those who do it the hospital would be obliged to provide board and lodging, or the equivalent in money, besides the regular wages. Under what other system then could an equally efficient class of workers be secured in the same systematic way, giving a full nine hours' service daily, and receiving financially less than the wardmaids? It is understood that the equivalent is to be made up by the education given. Is it not then a most serious responsibility on the part of such hospitals or training schools to see that education is made as complete as possible? After much practical experience I maintain that no such course of education can be thoroughly given in one year, but yet I find that very many schools limit their didactic teaching to the first year, and make the second year's work of a purely practical nature, and divide it between hospital work and private duty. It is absolutely necessary that class work and lectures should be carried on through the second year as well, and if this is done, then private nursing outside of the hospital is out of the question, as such interruptions would seriously interfere with any systematic teaching. I also hold that it is necessary to have the pupil under the daily observance and criticism of her teachers. This is impossible if she leaves the hospital for private duty, and one of two things must be true, viz., either she is as yet unfit to be entrusted to do her work without some supervision, or else, if she is really capable of doing this work in the second year, she should not be held by the school at all. In the latter case, why not make the term of pupilage only one year and graduate her? There is another side to this question, that of justice to the patient, but as this does not really come under the head of the standards we are just now discussing, we will pass it by.

### Everybody's Opinion.

*[Correspondence on all subjects is invited, but we cannot in any way be responsible for the opinions expressed by our correspondents. No communications can be entertained if the name and address of the correspondent is not given, or unless one side of the paper only be written on.]*

### AN ASSOCIATION OF NURSES FOR THE INSANE.

"A Matron" writes: Some years of experience in asylum work renders me much interested in a recent suggestion in The Hospital that there should be some association for all nurses employed in the care of the insane. The hospital nurse of to-day rightly gains much consideration for her present and future welfare, and surely something might be done for those who spend their time and energy in such important work as the charge of mental cases. I should be very glad if suggestions as to the best method of starting an association could be put forward by the readers of The Hospital.

### SHOULD NURSES BE A DISTINCT CASTE?

"An Old-Fashioned Nurse" writes: Many may think this a strange question, though anyone who notes the direction in which nurses are now drifting, or being driven, will see that it is time that it be seriously asked and answered for good or ill. For myself, I believe that for any body of servants to hold themselves unnecessarily apart from those for whose service they exist can only be "ill." With soldiers it means militarism, with ministers of religion, priestcraft; with all close corporations the interests of their own small part, rather than those of the general good. If nurses learn to consider themselves nothing but nurses, will they not be likely to think of their patients merely as "cases," instead of human beings with infinitely-varied capacities of learning the awful mystery of suffering? I am impelled to write this by the growing disposition among both nurses and the public to consider them as a class of beings apart from other womanhood, instead of being what they should be—women pre-eminently fitted by keenest sympathy to minister to the woes and needs of humanity. Your late strictures on street-sweeping skirts and jingling chatelaines are justly deserved, and will, I hope, be taken to heart by the offenders; but I fear those absurdities will not cease until nurses, like other Englishwomen, are content to keep their "shop" apparel for use in the "shop" alone. A nurse's uniform should be fitted for nursing *work*; the ordinary conventional outdoor garb is equally unsuited for work or play. A woman could neither ride, row, nor play tennis in it; whilst a woodland ramble would probably reduce it to tatters. The one thing for which it is fitted is to attract observation in the streets—a thing which the best type of women, whether nurses or not, would rather avoid! Only nurses engaged in district work require out-door uniform, and that should be of a description to promote heath, comfort, and economy, instead of ignoring all three, as is too often the case. For a woman who is supposed to be an evangelist of hygiene among the poor to wear a garb which scarcely varies whether the thermometer stands at 8 degrees or 80 degrees, and with a headgear which is next to useless as a protection from sun or wind, seems at least inconsistent. Let nurses remember that the reason of their existence as such is *nursing work*, and let them dress accordingly, reserving the gayer apparel in which most women love to indulge occasionally, for fitting times and circumstances, and not attempt an incongruous mixture of things essentially different.

### Church of England Sanitary Association.

The Church of England Sanitary Association held its first annual meeting at the Church House, Westminster, under the presidency of Dr. Norman Kerr. The adoption of the report for 1892 was moved by the Rev. Septimus Buss, seconded by the Rev. W. J. Hocking, and carried unanimously. The Rev. J. R. Byrne, H.M. Inspector of Schools, read a paper on "The Effects of Posture on the Health of School Children." Papers were also contributed by Dr. Robson Roose on the "Prevention of Cholera," and by Mr. F. W. Lowndes, M.R.C.S., on "Swearing by Holding up the Right Hand instead of Kissing the Book."

### Meetings.

### THE R.B.N. ASSOCIATION.

A banquet was given on the 17th inst. at the Hotel Metropole to celebrate the recent grant of a charter to this association. Sir William Savory occupied the chair, and the gathering included Sir James and Lady Crichton Browne, Sir Joseph Fayrer, Sir Trevor Lawrence, Mr. and Mrs. Brudenell Carter, Sir Edwin and Lady Saunders, Sir Dyce and Lady Duckworth, Sir Spencer Wells, Mrs. Fawcett, Mrs. Bedford Fenwick, and Miss Loch, of the Indian Army Nursing Service, who returned thanks for the army.

### Where to Go.

Floating Bazaars will be held at Great Yarmouth or at Gorleston on August 9th and 10th, and at Lowestoft on August 15th and 16th, in aid of the funds of the Mission to Deep Sea Fishermen. Contributions in goods or money should be sent to Bridge House, 181, Queen Victoria Street, London, before August 3rd.

### International Nursing Congress, Chicago.

(By Our Special Commissioner.)

### III.—DISTRICT NURSING.

Miss A. Hughes, Superintendent of the Metropolitan and National Nursing Association, read an excellent paper on the "Queen's Institute," which excited a great deal of interest. Then came one by Miss C. E. Somerville, Superintendent of the Lawrence General Hospital, Massachusetts, on "District Nursing in America." This was followed by an exhaustive paper by Mrs. Dacre Craven on "District Nursing in England." These three papers were among the most interesting of all those brought before the Congress. Mrs. Dudley commenced the discussion by inquiring how much time a nurse was allowed to give to each case in England. Miss Hughes explained that the time allowed varied according to the nature of the work to be done; much time was sometimes occupied where, in addition to bed-making and the preparation of food, the nurse had to wash the patient thoroughly. As a matter of routine it was usual to allow "half an hour for a man and three-quarters for a woman because of her hair." The average time was half an hour for chronic cases, and a maximum of an hour for those which presented special difficulty. Miss Wakem doubted the possibility of a district nurse making 21 visits in eight hours, as represented by Miss Somerville, for, in Chicago, the average was not more than eight or nine cases per diem. Miss Somerville explained that in Boston the city was divided into districts of reasonable proportions, a doctor and a nurse being assigned to each, which plan reduced the distances to a minimum. Sometimes a nurse had four cases in one street, and in such circumstances 15 minutes would be about the average time given to a case. A Boston district nurse pays on an averrge 16 or 17 visits in the day.

#### Self-Supporting and Free Associations.

In Philadelphia the aim has been to make the work of the District Nurses' Association self-supporting, or as nearly so as possible. Each patient is induced to pay something to prevent the possibility of pauperising, and Miss Bruebaker declared the system to be a success. Mrs. Dudley pointed out that most district nurses' associations were supported by voluntary contributions, which include gifts from societies and churches, and donations from football and athletic clubs, balls, and entertainments. It was the practice in Chicago for wealthy ladies to undertake the entire cost of one district nurse. Sometimes a capital sum was given, but more frequently the lady contributed a yearly sum sufficient to cover the entire cost of one nurse. Miss Wakem stated that in Chicago one nurse was permanently endowed, and six were supported on the annual system. A church sometimes undertakes the entire cost of a district nurse supplied by the association, a practice which might be followed with advantage in England. Mrs. Dudley informed Mr. Burdett that it cost £180 (900 dols.) to maintain one nurse for a year in Chicago. £3,000 (15,000 dols.) would provide an income sufficient to maintain a nurse permanently. In most cities it was customary for the street car companies to grant free passes to district nurses, but Chicago is an exception to this general rule, and charges each nurse full fare whenever she uses the cars. It seemed to be the general opinion that every district nurses' association should be so organized as to enable it to undertake cases where a patient should pay something for the nurse's services, in addition to the free cases. It was further thought objectionable for the nurse-training schools to supply nurses for district work because of the frequent changes which ensued, and also because undergraduates, *i.e.*, non-certificated or pupil nurses, were frequently supplied, who had not sufficient experience or training to enable them to do the work properly. The district nurses' associations of Boston and Kansas City have no endowment.

### Day and Night Calls.

Although night-calls were attended to by most associations, they found it necessary to provide that a nurse should not attend to a night-call unless it was accompanied by a written order from the doctor stating the case to be urgent. Miss Wakem stated that, when she was in charge of the cottage settlement of the Chicago District Nursing Association, during one month, out of 77 night-calls only two were found to need the services of a nurse. For instance, if a messenger came and stated that his mother was very ill, and did not produce an order from a doctor, the statement was entered in the visiting book, and the nurse in whose district the patient resided called the next day. This rule was enforced at Philadelphia as well as in London. There can be no doubt of the necessity of protecting nurses from calls at all hours of the night, when rest must be taken, especially as experience proves that all sorts of trivial questions are asked, and that, as in Miss Wakem's case, some 98 per cent. of all night cases will keep till next day.

### Homes for District Nurses.

In the United States the district nurses are usually housed in lodgings, as the funds are not sufficient to enable the committees to take a house. Miss Somerville stated that her committee thought it was just as well for nurses to live separately, because, when they got to their homes, they did not talk "shop." Their surroundings were better, and the "lodgings" system worked well in every way. On the other hand, Miss Bruebaker stated that the nurses were accommodated in a Home in Philadelphia, although the cost of such accommodation was greater than that of lodgings. Miss Hughes explained the working of the district homes in London, with their local boards of managers. It seemed to excite a good deal of surprise that local contributions in London should amount to so large a sum, as this system of support has not yet become popular in the United States. Miss Wakem thought it would be greatly to the advantage of the nurses in many ways if they were to establish a home in Chicago.

### Responsibility for Disabled and Sick Nurses.

We were always of opinion that every employer recognised the duty of providing, to some extent, for nurses who were struck down by illness in the discharge of their arduous duties. Mrs. Swanton, however, saw no reason why an association should be responsible for those whom it employed. Miss Hughes explained that the Royal National Pension Fund for Nurses by its scheme of affiliation had materially assisted the district nurses' associations, who were enabled by its means to make adequate provision for their nurses at a reasonable cost. When a nurse fell ill in the course of her duties she was taken charge of. Sometimes nurses were sent to the seaside and sometimes to a convalescent home, until they were able to resume their work. One nurse who hurt herself when on duty had her salary continued and was supported by the association, as she was without friends, and, had she been permanently disabled, she would have been provided for by the Royal National Pension Fund for Nurses. Miss Somerville said that the Boston Association also provided for its nurses under similar circumstances. In the United States it was customary for nurses to form alumni associations, and to tax themselves for the support of any of their number who might fall ill and need assistance in the course of the year. Miss McAvoy pointed out to Mrs. Swanton that some provision was absolutely required by disabled district nurses, because their chances of saving were so slender. A district nurse had little opportunity to lay by, and the work was one of much self-sacrifice, seeing that the remuneration was infinitely smaller than a nurse could earn by private work. Mrs. Swanton, who seemed, however, to be opposed to all provision for disabled nurses, declared that any woman who could not afford to sacrifice herself to district nursing should confine herself to private nursing. Miss Somerville stated that in the case of a devoted nurse, who had worked faithfully for years and then became ill, the President of the Association was thinking of starting a private subscription to help her, though he did not consider there was any obligation upon the Association to take any such steps. The Editor of The Hospital receives frequent requests from nurses in the United States, who become anxious because there is no Pension Fund in that country into which they can put their savings, and to which they can look should they be struck down by illness or permanently disabled. It is well known that a sum of over £20 000 ($100,000) is available for starting a Pension Fund in the United States, but that a few active spirits have so far prevented the founding of such a fund, because they decline to receive any assistance or help from well-wishers, who may be able and willing to give both time and money to so good a cause. Is it not time that the superintendents, who have now formed themselves into an association, should take this matter up, and place themselves in communication with Mr. Pierpont Morgan on the subject?

### The Missionary versus the Professional Spirit.

An interesting discussion arose on the question "Which class of nurses are more efficient, those actuated by the missionary spirit, or those by the professional spirit?" Miss Somerville considered that the missionary spirit was absolutely essential. The missionary spirit meant endurance, while the professional spirit caused women to throw up the work in a short time as too hard. On the other hand, a strong professional spirit was an advantage, as it arose from the deep interest which a nurse took in her cases, so that a leaven of the missionary spirit was necessary to reconcile her to the duty of properly attending to chronic and troublesome patients whose cases are usually uninteresting. There can be no doubt that the missionary spirit largely prevails amongst American nurses, who do a great many acts of kindness by attending patients without charge, and by subscribing liberally in support of members of their body who fall ill or become disabled. This is the true missionary spirit, and those nurses who possess it, and most nurses we believe do, are sure to have the right professional spirit also, which will lead them to regard their calling as a profession demanding at their hands the maximum of zeal and devotion.

### Alumni Associations.

Miss Hampton, the President, gave expression to the following views on this subject: "I hope in the course of time we shall be able to evolve alumni associations, an American Nurses' Association, and a superintendents' convention. The only way I can see to accomplish all these is not to be in a hurry. I think we have accomplished a great deal already at our meetings here in Chicago, and they show that later on, if we set about it in the right way, we shall succeed in attaining all our objects. Superintendents as the heads of training schools have a great deal of influence, not only among their pupil-nurses, but amongst the graduate nurses too. Until we can get the superintendents united upon a common platform we cannot expect our nurses to work, and to unite, and to be as successful as they must be later on, when we hold ideas in common. There is one thing we can accomplish immediately as the result of the papers, and that is to start alumni associations in connection with every training school in the country. They should be as nearly alike as possible. Of course, some of the details must be different to meet special demands, but let us resolve to get as much uniformity as possible, and then we can go forward to the superintendents' convention. I do not think superintendents should take an active part in alumni associations. These associations should be taken up and worked out by the members of the training-schools themselves. If they make mistakes they will find out by such mistakes the better way. Now do not be in a hurry, because, until nurses get their alumni associations into good working order, it will be impossible to organise a national association, which, to be a success, must adequately represent the nurse training schools, the hospitals and the nurses themselves. The training-schools can only be represented through the superintendents and board of managers, and the nurses can only be represented through their alumni associations. Hence, the first work to be done is to organise the alumni associations and the superintendents' convention. The nurses must take care of the alumni associations; the superintendents will take care of the convention; and then we will all take care of the American Nurses' Association."

At the conclusion of Miss Hampton's address, a meeting was held, at which we understand that a superintendents' convention was formed, which promises to do much for trained nurses in the United States. Miss Nightingale's paper on Friday morning was received with great satisfaction, and the whole Congress, which was largely attended, grew more and more enthusiastic every day. It was a complete success, and more practical and instructive papers were read than have ever been presented at any similar congress.

### British Columbia.

### A HOSPITAL FOR THE INDIANS.

(Communicated.)

A week's sick leave to spend at the Mission House, gave an opportunity to inspect an Indian hospital which the Bishop is building at Lytton. The place itself is hemmed in with mountains, but through the gaps between these a perpetual current of wind is admitted. I got out of the train into a high wind, and was speedily transformed from a neat city nurse into a mere bundle of streamers. Climbing down a slope, I came to the Mission House, just a large cottage with three rooms below, and two attics above, the latter being the bed-rooms. All the down-stair rooms opened into each other, and were sitting-room, kitchen, and surgery. The last is the scene of amusement sometimes. All day long the Indians come in for medicine. They cannot speak English, so an Indian girl interprets, or else the resident priest. The patients generally complain of aches and pains all over, and when ill come straight off to the white man for some medicine, returning home with every faith in the physic received.

This place is the centre of the Indian reserve, and one village is wholly Indian, having its own church. Mr. Small, the resident priest, has learned enough of their language to preach to and teach them. The Bishop has got a grant from Government for building a small hospital next door to the Mission House, and has asked to have trained nurses provided from St. Luke's Home, at any rate for the present.

Next month, therefore, the hospital will be formally opened at Lytton, which is not a town or even village, but only a street with houses on each side, which can be traversed in a few minutes. It is excessively cold in the winter, and quite dependent upon Vancouver for supplies. There is a train once a day going through to Montreal in the evening; and in the morning the Pacific express, going to Vancouver. They only stay a few moments to pick up or to leave stray passengers, and to drop the mail.

On Sunday we all went to the Indian church, where the congregation consisted of about forty women, and possibly more men, the women all seated on the left and the men on the right. The Litany was sung in the Indian language, and then a Confirmation service was held, followed by the Holy Communion.

The Indians were most reverent, and the watchman, or head man, wearing a badge on his arm with a Maltese cross worked on it, was responsible for their morality and good behaviour. They all aspire to dress like Europeans, and grotesque, indeed, was the effect of men in shirt sleeves, blankets, old coats, &c. One man was quite fashionable in black trousers, velveteen coat (quite new), and a pink silk tie. Amongst the women white or velvet dresses were seen, and also old skirts with merely a shawl or blanket round the shoulders.

If anyone feels disposed to help us with the new little hospital they can send contributions to the "Indian Hospital, Lytton, B.C.," whether in money or goods, or addressed to the Bishop of New Westminster, B.C. One reason why the English should help is our having promised to receive accidents occurring among the white men. A mine is being worked just across the river by a company, and there are several English gentlemen there, so that we expect to benefit the miners considerably as well as the clergy.

The Bishop got up a concert one evening, and all the mining people and train hands took tickets, about 130 guests being present. Two strange Englishmen handed in a sovereign each and an old Australian traveller gave another, and a Bradford friend a third, so we had £20. Next day a few more subscriptions came in, making £25 in hand towards furnishing.

We hope to enlist the sympathies of the Vancouver nurses and get them to give three months each to the new Indian hospital. Lytton is a very healthy place, and the walks outside it are charming.

### Notes and Queries.

### Queries.

(160) *Dolls.*—What size and style of doll is best for dressing in uniform ?—*Ladies' Companion.*

### Answers.

(160) *Dolls (Ladies' Companion).*—Not too large, and any style which commends itself to the purchaser. Of course it is always easier to dress a well-made doll than a clumsy one.

### For Reading to the Sick.

### TRIFLES.

So many of us find our lives failures because we are impatient and not contented with the present moment. If we are healthy and have pleasant surroundings, we are not happy, for we long after something unattainable, or which would harm us if we had it. The well-known Eastern story is quite to the point. Aladdin, by the aid of his wonderful lamp, surrounded the beautiful Princess his wife with every luxury that heart could desire. Gold and jewels shone everywhere, precious silks and stuffs covered the couches, and lovely pictures hung on the walls around. Beautiful birds, plants of every hue, sparkling fountains, and ravishing music, joined in making the Palace a home of delight. But a wicked spirit whispered in the Princess's ear, "This place is not perfect; it should have a roc's egg hanging from the middle of the ceiling." Now the roc's egg was just the one thing which Aladdin ought not to have procured, and in trying for it (like the forbidden fruit in Paradise), he brought ruin on himself with its possession.

How often we lose our happiness in the same way. We despise the trifles which lie around us, forgetting that "trifles make the sum of human life." Our happiness depends on them, and how we take and use them. "There's many a gem in the path of life, which we pass in our idle pleasure" far richer than the "jewelled crown," or the "miser's treasure," which excite our envy. It may be only the love of a little child, or a mother's prayer to heaven, or a beggar's grateful thanks for a cup of cold water given to him; yet all these things can make our lives beautiful, if we will not listen to the wicked spirit, who says to us, "You ought to be thought more of, and your wishes considered by those around you," or "You ought to be richer or have greater opportunities for pleasure." And if we are sick, perhaps the little demon of discontent says "Your bed is in the worst place in the room, if you were only a little more to the right or the left you could see all that is going on in the ward and would be amused," while really you have the snuggest, nicest spot possible; light and airy, with no possibility of draughts. How sad it is to spoil our lives in this way. We think of ourselves till we conjure up imaginary grievances, like the poet's little son who could give no better reason for disliking a new charming home than that it had no weather-cock on the roof as his old one had. Some are naturally more cheerful and contented than others, but good spirits to be lasting must be strengthened by religion. To know and be satisfied that our Heavenly Father sends us alike pleasures and disagreeables, that blessings come from Him sometimes in disguise, and that we should try to see through the veil which covers them, are truisms on which depend our comfort in life. Believe me,

There is many a rest on the road of life,
  If we would only stop to take it,
And many a tone from the better land,
  If the querulous heart would make it.
To the sunny soul that is full of hope,
  And whose beautiful trust ne'er fadeth,
The grass is green and the flowers are bright,
  Though the wintry storm prevaileth.

### Appointments.

Tetbury Cottage Hospital.—Miss Gertrude B. Thorold has been appointed Matron of the Tetbury Cottage Hospital. She was trained at the Alexandra Hip Hospital, Queen's Square, and the Sussex County Hospital, and has had several temporary appointments. Her testimonials are good, and we wish her every success.

### Minor Appointments.

Chesham Cottage Hospital.—Miss Elizabeth A. Craggs has been made Nurse of the Chesham Cottage Hospital. She was trained at the Cheltenham General Hospital, and afterwards worked at the Birmingham General Hospital for five years, and has our best wishes for her success in her new work.

### The Book World for Women and Nurses.

[We invite Correspondence, Criticism, Enquiries, and Notes on Books likely to interest Women and Nurses. Address, Editor, THE HOSPITAL (Nurses' Book World), 428, Strand, W.C.]

DOMESTIC ECONOMY FOR STUDENTS AND TEACHERS. By ADA COLLEY. (Simpkin, Marshall, and Co.)

This moderate-sized book aims at giving instructions on a great variety of very important subjects, namely, Food, Clothing, Washing, the House, Furniture, Ventilation, Income, Hygiene, and Health. It is hardly possible that so many things can receive uniformly able treatment. Food in itself offers a large field to any writer, and Miss Colley has taken a kind of "reading made easy" method, hardly befitting the dignity of one who aspires to teach pupil teachers. "Veal is the flesh of the calf or young cow," is by no means a solitary instance of this. We cannot agree with the statement that the children of the working classes are generally sturdy and strong, although this may be true of the middle classes with whom they are here coupled. The demand for increasing accommodation in Children's Hospitals, Homes, and institutions for chronic and incurable children points to a less satisfactory condition. The pages devoted to clothing give much useful information, though Miss Colley's theory that silk is better than flannel underclothing will not receive universal acquiescence. For persons who can afford the luxury, and who fancy they cannot wear flannel, silk is doubtless an agreeable alternative, but in England at any rate there are very few months in the year during which woollen underwear is not the safest and best. There is no reason it should be "thick and bulky," as it is called in this little book, for wool is manufactured into the finest and daintiest of fabrics, as well as the coarsest. Laundry work in Board Schools is well described, and seems likely to uphold its present popularity, the pretty illustrations adding considerably to the attractions of the washing section in the book. The dwelling, selection of a site, &c., are also well laid before the reader. It is a little difficult to understand for which class the house itself is designed, as we are told that the bed-rooms should be large, lofty, and airy, which presupposes a somewhat capacious mansion, yet the sitting-room is to be a "parlour," which two tables, "a cheffonier and a chimney glass" will adequately furnish when the inevitable upholstered "suite" is installed. The dining-room or general living-room is to have horsehair or leather furniture; and again we must differ from the writer, for surely horsehair seats and cushions may be regarded as obsolete, and bentwood, or some such chairs, are surely cleaner and more economical than the kinds recommended. The parlour is supposed to be used on Sundays more than other days, and we fear the housewife will never have very much time for contemplating her cheffonier on any day in the week, if she fulfil all the duties laid down for her. The washing is to be done at home, and no one can doubt the economy of this practice if the mother has strength and knowledge of this heavy work. We are sorry to read that the bed-room windows should be closed at dusk, for we fail to see how ventilation will be secured through the night even in the "large and lofty" bed-rooms, which, by the way, are all to be cleaned on a Thursday morning, in addition to the cooking, &c. In the chapter on ventilation the opening of bed-room windows during the day is also considered sufficient, although other modes for the ingress and egress of air are not omitted. Income and expenditure receive several pages of practical advice, and so does hygiene, though both subjects must be rather beyond the comprehension of those who have to be told that "veal is the flesh of the calf." The general remarks on health can be cordially commended to all readers, and so can those on the sick-room, but not so the chapter on General ailments and How to treat them. The last, if carried out as here set down, would be as distinctly displeasing to the doctor as dangerous to the patient. To lay down as a general rule that measles and scarlet fever cases are to be treated by baths, without reference to the medical adviser, is simply appalling to anyone who knows the risks which might attend such unauthorised proceedings. Why are "scarlet fever and scarletina" spoken of? the latter word, usually spelt "scarlatina," being merely the technical term for the former. The treatment of diphtheria and ringworm is given, but we venture to think that it would have been wiser for Miss Colley to impress upon her readers the need for immediate medical treatment and skilled nursing in the former terrible disease. A dangerous piece of advice is given with regard to convulsions, for we read "the child should be plunged into mustard and water as warm as the hand can bear it." It might, indeed, be "counter-irritation" to add an extensive scald to the sufferings of a convulsed child, but it is hardly a justifiable proceeding. A woman whose hands are accustomed to many degrees of heat and cold in the course of her daily avocations can easily endure contact with mustard and water which it would be surely great cruelty to apply to a poor little child's delicate and sensitive skin. In the chapter headed "Common Accidents and their First Aids," we find many useful hints, but again the general mistake occurs of confusing "first aid" with "treatment," unprofessional people always find the distinction a difficulty, and if they allow themselves to be guided by the instructions given in the pages before us they will find "confusion worse confounded" is their portion. In burns the application of flour first and oil on top, would be decidedly bad for serious injuries although it might not be inappropriate for a mere scorching of the skin. Like many text books this one deals with the wounds rather than the wounded, omitting all mention of the need for immediately securing warmth for the patient and for taking steps to combat the shock which is such a terrible accompaniment of bad burns or scalds. To syringe an ear, into which some foreign body has entered is certainly risky; the last words of the paragraph should be the first, for if the advice they embody were realised before and not after syringing, it would be better for the sufferer "a medical man should be called, as the drum of the ear is too delicate an organ to be trifled with," and assuredly a syringe in untrained hands is an instrument capable of entailing irremediable disaster. If a book on Domestic Economy compiled to aid students and teachers, includes directions on "first aid," it is desirable that the instructions should be accurate and such as can be advantageously followed out by persons without surgical knowledge or training in skilled nursing, otherwise they are worse than useless.

---

The Ladies' Sanitary Association, 22, Berners Street, W., has added another excellent tract to its popular series. If "General Rules for the Guidance of Maternity Nurses" were read by every married woman, many useful hints would be received. The modest price of this useful pamphlet is one halfpenny, but most purchasers will, we think, be inclined to invest in numerous copies to distribute to their poorer neighbours.

